# Transcriptional Regulation of an Insulin-Sensitizing Adipokine Adipolin/CTRP12 in Adipocytes by Krüppel-Like Factor 15

**DOI:** 10.1371/journal.pone.0083183

**Published:** 2013-12-16

**Authors:** Takashi Enomoto, Koji Ohashi, Rei Shibata, Takahiro Kambara, Yusuke Uemura, Daisuke Yuasa, Yoshiyuki Kataoka, Megumi Miyabe, Kazuhiro Matsuo, Yusuke Joki, Satoko Hayakawa, Mizuho Hiramatsu-Ito, Masanori Ito, Toyoaki Murohara, Noriyuki Ouchi

**Affiliations:** 1 Department of Cardiology, Nagoya University Graduate School of Medicine, Nagoya, Japan; 2 Department of Molecular Cardiology, Nagoya University Graduate School of Medicine, Nagoya, Japan; University of Tor Vergata, Italy

## Abstract

Obese states characterized by chronic inflammation are closely linked to the development of metabolic dysfunction. We identified adipolin/CTRP12 as an insulin-sensitizing and anti-inflammatory adipokine. Although obese conditions down-regulate adipolin expression, its molecular mechanism is largely unknown. Here we show that the transcriptional regulator Krüppel-like factor (KLF) 15 is involved in the regulation of adipolin expression in adipocytes. White adipose tissue from diet-induced obese (DIO) mice showed decreased expression of KLF9 and KLF15 among several KLFs, which was accompanied by reduced expression of adipolin. In cultured 3T3L1 adipocytes, treatment with TNFα significantly reduced the mRNA levels of KLF9, KLF15 and adipolin. Adenovirus-mediated overexpression of KLF15 but not KLF9 reversed TNFα-induced reduction of adipolin expression in adipocytes. Conversely, gene targeting ablation of KLF15 attenuated adipolin expression in adipocytes. Expression of KLF15 but not KLF9 enhanced the promoter activity of adipolin in HEK293 cells. Pretreatment of 3T3L1 adipocytes with the JNK inhibitor SP600125, but not p38 MAPK inhibitor SB203580 blocked the inhibitory effects of TNFα on adipolin and KLF15 expression. These data suggest that adipose inflammation under conditions of obesity suppresses adipolin expression via JNK-dependent down-regulation of KLF15 in adipocytes.

## Introduction

Obesity is closely associated with the development of various metabolic disorders including insulin resistance and type 2 diabetes [1]–[3]. Recent evidence indicates that chronic low-grade inflammation contributes to the pathogenesis of numerous obesity-related diseases such as atherosclerosis and insulin resistance [4]–[7].

Adipose tissue secretes numerous biologically active proteins, also known as adipokines. Many of the adipokines, including TNFα, MCP-1 and IL6, promote inflammation and metabolic dysfunction, whereas a small number of adipokines have beneficial actions on inflammatory processes and metabolism [5], [8], [9]. We have demonstrated that adiponectin exerts protective functions against obesity-related diseases through modulation of inflammatory responses [10]–[16]. Recently we found that adipolin/C1q/TNF-related protein (CTRP) 12 functions as an anti-inflammatory adipokine that is abundantly expressed in fat tissue [17]. We also showed that systemic administration of adipolin improves insulin sensitivity in diet-induced obese (DIO) mice [17]. Consistent with our findings, another report demonstrated that adipolin ameliorates glucose metabolism in obese and diabetic mice [18]. Plasma adipolin concentrations are reduced in rodent models of obesity [17], [18]. Adipolin expression is also reduced in fat tissue in obese mice [17], [18]. Furthermore, adipolin expression is suppressed in cultured adipocytes by the pro-inflammatory stimuli [17]. Thus, it is plausible that adipose tissue inflammation in obese states is involved in the regulation of adipolin expression. However, the precise mechanism of how obese states reduce adipolin expression in fat tissue has not been fully clarified.

Krüppel-like factors (KLFs) are a family of zinc-finger transcription factors that recognize GC-rich elements and CACCC boxes [19]. KLFs are broadly associated with disorders including obesity, inflammatory conditions and metabolic dysfunction [20]. Among KLFs, the expression levels of KLF9, KLF10, KLF13 and KLF15 are increased during adipocyte differentiation of 3T3-L1 cells [21]. It has been shown that KLF15 plays a crucial role in regulation of adipocyte differentiation [21]. KLF9 is also reported to act as a pro-adipogenic transcription factor [22]. Adipolin expression is up-regulated upon adipocyte differentiation in vitro [17], [18]. These findings allowed us to speculate the potential involvement of KLFs in regulation of adipolin in adipocytes. Here, we sought to dissect the mechanism of obese conditions regulate the adipose expression of adipolin.

## Materials and Methods

### Materials

Recombinant TNF-α protein was purchased from Sigma. SB203580 and SP600125 were purchased from TOCRIS and Wako, respectively. An antibody against FLAG was purchased from Cell Signaling Technology.

### Cloning of mouse KLF15 and KLF9

Full-length mouse KLF15 cDNA (accession number: NM_023184.3) and KLF9 cDNA (accession number: NM_010638.4) were cloned by PCR using cDNA from mouse epididymal fat tissue, fused with FLAG epitope at the C-terminus, and subcloned into the pShuttle mammalian cell expression vector (Qbiogene). The pShuttle vector expressing KLF15 or KLF9 was transfected into HEK293 cells by using Lipofectamine LTX (Invitrogen) according to manufacturer's instruction. Cells transfected with empty vectors (mock) were used as a negative control.

### Animal model

Control C57BL/6J, high fat (HF) diet-induced obese (DIO) in a background of C57BL/6J were purchased from Charles River Laboratories. C57BL/6J mice at the age of 4 weeks were maintained on a HF diet (Research Diets, Inc. D12492, 60% fat) or a normal diet (CLEA CE-2, 4.8% fat, wild-type mice) for 12 weeks. Study protocols were approved by the Institutional Animal Care and Use Committees at Nagoya University. Our study conformed to the Guide for the Care and Use of Laboratory Animals published by the United States National Institutes of Health.

### Cell culture

Mouse 3T3-L1 cells (ATCC) were maintained in DMEM with 10% fetal bovine serum (FBS) and differentiated into adipocytes by treatment with DMEM supplemented with 5 µg/ml of insulin, 0.5 mM 1-methyl-3-isobutyl-xanthin, and 1 µM dexamethasone [23]. At day 8 after differentiation, 3T3-L1 adipocytes were treated with SB203580 (10 µM), SP600125 (10 µM), TNFα (10 ng/ml) or vehicle for 24 h.

### Transfection with siRNA targeting KLF15 in 3T3-L1 adipocytes

3T3-L1 adipocytes were transfected with siRNAs targeting KLF15 or unrelated siRNAs at 20 nM for 48 h (Thermo Scientific, ON-TARGET plus SMART pool, Cat. No. L-059453-01) by using Lipofectamine RNAi MAX reagent (Invitrogen) in a cell suspended condition after trypsin treatment according to the method by Kilroy et al [24].

### Construction of adenoviral vectors

The adenovirus (Ad) vectors expressing β-galactosidase (Ad-(-gal), Flag-tagged KLF9 (Ad-KLF9) and Flag-tagged KLF15 (Ad-KLF15) were constructed under the control of CMV promoter [25], [26]. 3T3-L1 adipocytes were transduced with Ad-(-gal, Ad-KLF9 or Ad-KLF15 at a MOI of 150 for 24 h. The media was then replaced with fresh DMEM, and cells were incubated for additional 24 h.

### Luciferase reporter assays

3T3-L1 adipocytes or HEK293 cells in 24 well or 96 well dishes were transfected with 1 µg or 200 ng of pGL3-basic vector (Promega, Madison, WI) containing a fragment of the mouse adipolin gene promoter (nucleotides −111 to −1 or −66 to −1 relative to the transcription initiation site) or empty pGL3-basic vector along with 200 ng of pShuttle expression vector containing mouse KLF9 or KLF15 cDNA, or the empty pShuttle vector (mock) in the presence of 10 ng or 4 ng of pRL-SV40 (Promega) by Lipofectamine LTX (Invitrogen) as described previously (5). After 48 h, the cells were lysed in Passive Lysis Buffer (Promega), and the lysates were assayed for firefly and Renilla luciferase activities with the use of a Dual-Luciferase Reporter Assay system (Promega). Promoter activity was determined as the ratio of firefly to Renilla luciferase activities.

### Quantification of mRNA levels

Gene expression level was quantified by real-time PCR (RT-PCR) method. Total RNA was prepared using a Qiagen kit. cDNA was produced using ReverTra Ace qPCR RT Master MIX (ToYoBo). PCR was performed with a Bio-Rad real-time PCR detection system using Thunderbird qPCR Mix (ToYoBo) as a double-strand DNA specific dye. Primers were: 5′-taccacgtccaaccgtgag-3′ and 5′-gtcatgtgggcatctgagag-3′ for mouse adipolin, 5′-TACAGGAGAAAAGCCGTACAAATG-3′ and 5′-TCATCAGACCGAGCGAACTTC-3′ for mouse KLF3, 5′-ATACAGGTGAACGGCCCTTTC-3′ and 5′-TCCGAGCGCGAGAACTTTT-3′ for mouse KLF9, 5′-TTCTCTCCAGCAAGCTTCGGA-3′ and 5′-TCACTCTGCTCAGCTTTGTCCC-3′ for mouse KLF10, 5′-CCTCACCCCTTTGGTAATAGGA-3′ and 5′-GCTGTGGACTTCTCAAGTTCGC-3′ for mouse KLF13, 5′-TACACCAAGAGCAGCCACCT-3′ and 5′-AACTCATCTGAGCGGGAAAA-3′ for mouse KLF15, 5′-AGGTTGGATGGCAGGC-3′ and 5′-GTCTCACCCTTAGGACCAAGAA-3′ for mouse adiponectin, and 5′-GCTCCAAGCAGATGCAGCA-3′ and 5′-CCGGATGTGAGGCAGCAG-3′ for mouse 36B4.

### Western blot analysis

Cell lysates of 3T3-L1 adipocytes were used for determination of overexpressed- KLF15 and KLF9, both of which are tagged with FLAG. Equal amounts of proteins were separated with SDS–PAGE. After transferring to PVDF membranes, Western blot analyses were performed with the antibodies against FLAG. ECL Western Blotting Detection kit (GE Healthcare) was used for detection.

### Statistical analysis

All data are expressed as means ±SEM. Differences were analyzed by Student's unpaired t test or one-way analysis of variance (ANOVA) with the Tukey post-test. A value of P<0.05 was accepted as statistically significant.

## Results

### Reduced expression of KLF9 and KLF15 in obese adipose tissue

**Figure 1:**
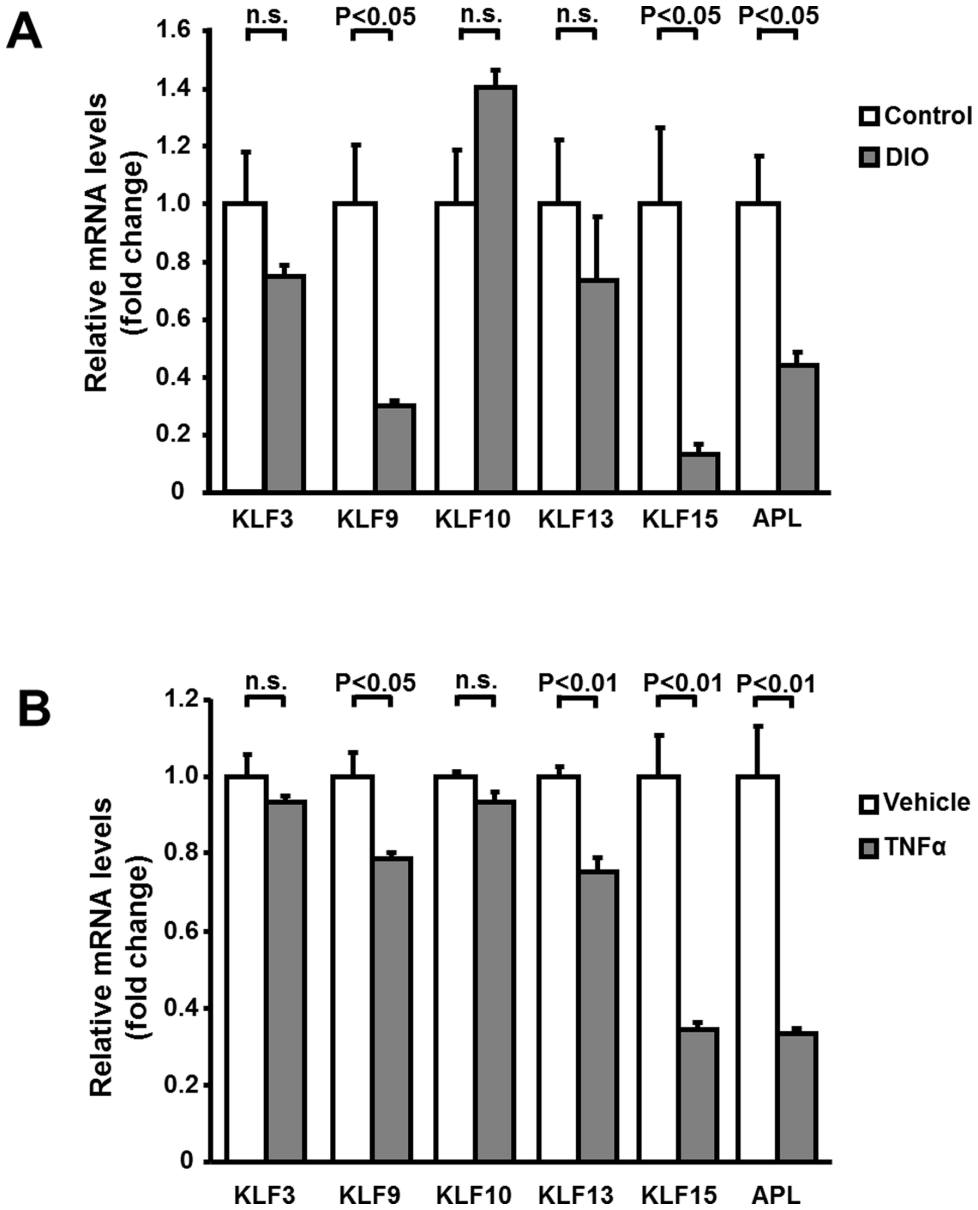
KLF9 and KLF15 are down-regulated in adipose tissue of obese mice and TNFα-treated cultured adipocytes. Quantitative RT-PCR method was used for measurement of mRNA levels. **A**, mRNA levels of KLF3, KLF9, KLF10, KLF13, KLF15 and adipolin (APL) in adipose tissues of control lean and high fat diet-induced obese (DIO) mice. N = 3–4 in each group. **B**, mRNA levels of KLF3, KLF9, KLF10, KLF13, KLF15 and adipolin (APL) in 3T3-L1 adipocytes. 3T3-L1 adipocytes were treated with TNF-α (10 ng/ml) or vehicle for 24 h. N = 3 in each group.

To investigate whether obesity modulates the expression KLFs in adipose tissue, the mRNA levels of KLF3, KLF9, KLF10, KLF13 and KLF15 were evaluated in epididymal adipose tissue of diet-induced obese (DIO) and control lean mice by RT-PCR methods. KLF9 and KLF15 mRNA levels were significantly reduced in adipose tissue from DIO mice compared with those from control mice (Figure 1A). The mRNA levels of KLF3, KLF10 and KLF13 did not differ in adipose tissue between two experimental groups. Adipolin mRNA expression was decreased in fat tissue in DIO mice compared with control mice consistent with the previous report [17]. To test whether the pro-inflammatory stimuli affects the expression KLFs in cultured adipocytes, 3T3-L1 adipocytes were treated with recombinant TNFα protein or vehicle. TNFα treatment significantly decreased the mRNA levels of KLF9, KLF13 and KLF15 in cultured 3T3-L1 adipocytes (Figure 1B). In contrast, TNFα did not change the expression of KLF3 and KLF10 in adipocytes. Adipolin mRNA levels were suppressed in adipocytes by TNFα in agreement with the previous report [17].

### KLF15 overexpression restores TNFα-mediated reduction of adipolin expression in adipocytes

**Figure 2:**
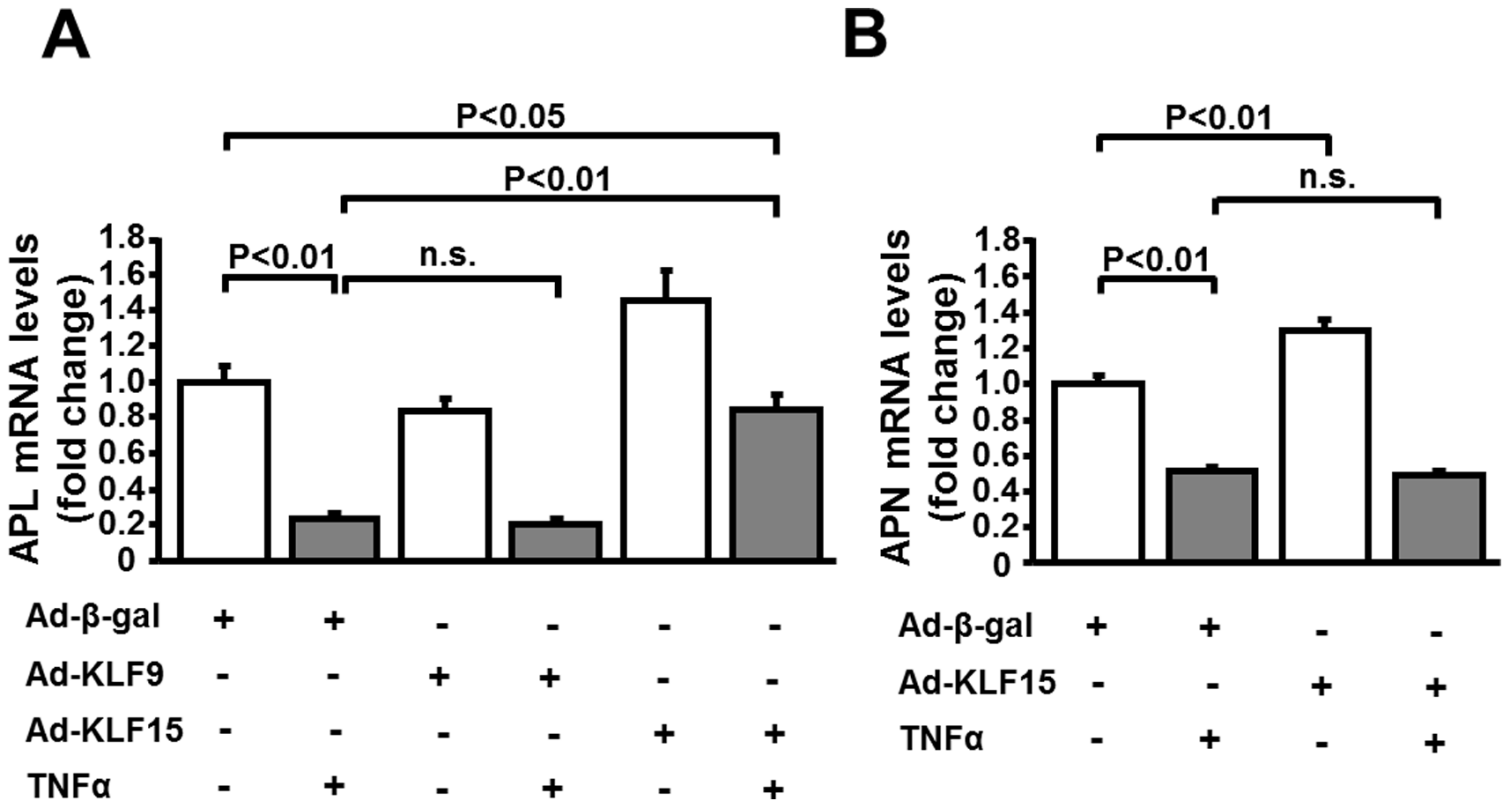
Overexpression of KLF15 rescues the reduction of adipolin expression caused by TNFα. Quantitative RT-PCR method was used for measurement of mRNA levels. **A**, Adipolin mRNA levels treated with adenovirus expressing KLF9 (Ad-KLF9), KLF15 (Ad-KLF15) or β-galactosidase (Ad-β-gal) at 150 moi for 24 h in 3T3-L1 adipocytes. 3T3-L1 adipocytes were treated with TNFα (10 ng/ml) or vehicle for 24 h. N = 3 in each group. **B**, Adiponectin mRNA levels treated with Ad-KLF15 or Ad-β-gal at 150 moi for 24 h in 3T3-L1 adipocytes. 3T3-L1 adipocytes were treated with TNFα (10 ng/ml) or vehicle for 24 h. N = 3 in each group.

To investigate whether KLF9 and KLF15 affect adipolin expression in vitro, 3T3-L1 adipocytes were transduced with adenoviral vectors encoding KLF9 (Ad-KLF9), KLF15 (Ad-KLF15), or control β-galactosidase (Ad-β-gal). Transduction of adipocytes with Ad-KLF15 led to a significant increase in adipolin expression (Figure 2A). Ad-KLF15 treatment reversed the inhibitory effects of TNFα on adipolin expression in adipocytes. In contrast, Ad-KLF9 had no effects on adipolin expression in adipocytes in the presence or absence of TNFα. These data indicate that KLF15 enhances the expression of adipolin in cultured adipocytes. We also examined the impact of KLF15 on another insulin-sensitizing adipokine adiponectin in vitro. Treatment of adipocytes with Ad-KLF15 resulted in a marginal increase in adiponectin mRNA expression (Figure 2B). TNFα treatment suppressed adiponectin expression in adipocytes, and this suppression was not affected by Ad-KLF15 (Figure 2B).

### KLF15 enhances promoter activity of adipolin in HEK293 cells

**Figure 3:**
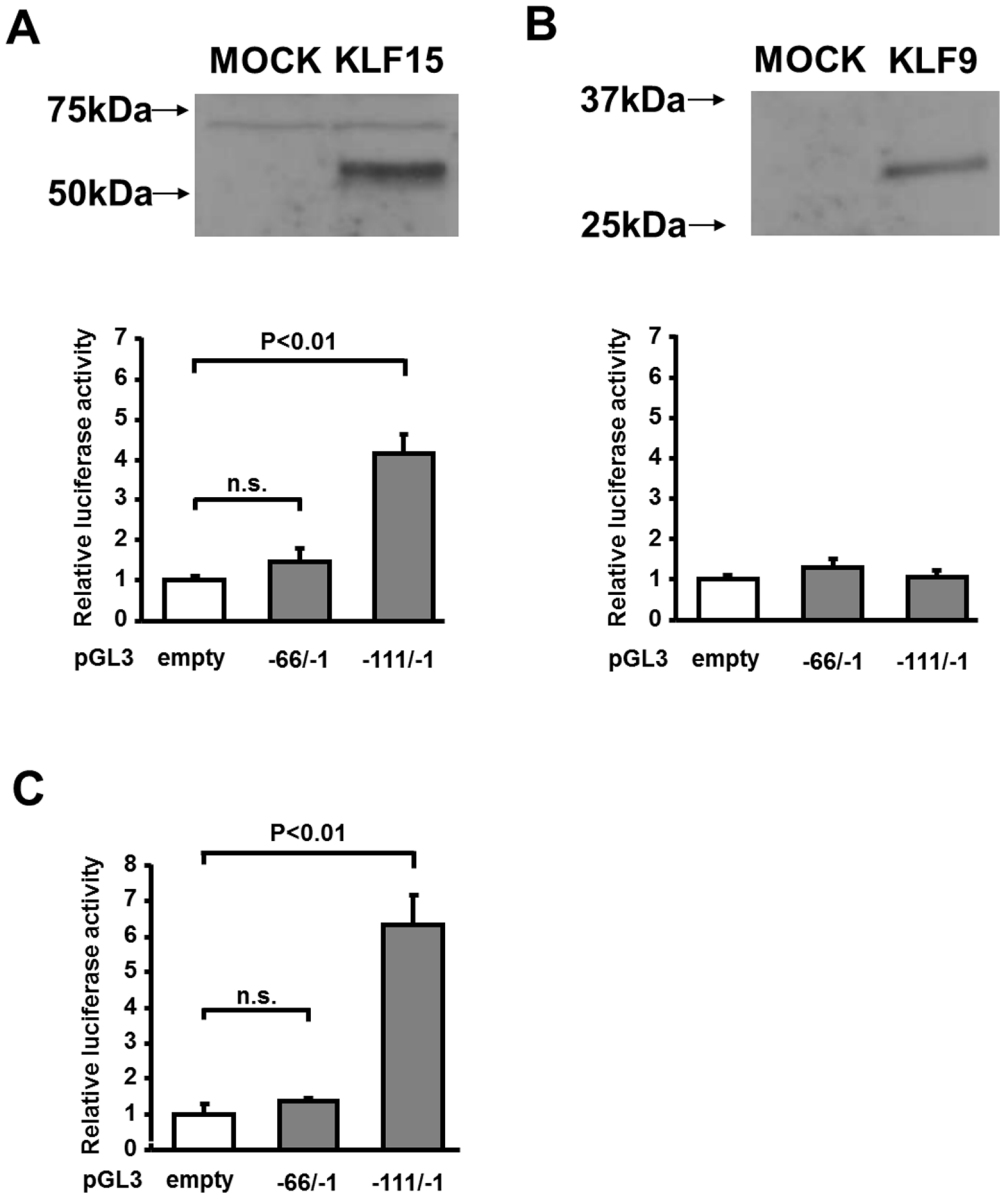
Expression of KLF15 augments the promoter activity of adipolin. **A and B**, Effect of KLF15 and KLF9 on the promoter activity of adipolin. Protein levels of KLF15 (A) and KLF9 (B) in HEK293 cells transfected with pShuttle vector expressing KLF15 tagged with FLAG, KLF9 tagged with FLAG or empty vector (MOCK). Expression of KLF15 and KLF9 was evaluated by Western blot analyses using anti-FLAG antibody. HEK293 cells were transfected with pShuttle vector expressing KLF15, KLF9 or MOCK, along with pGL3-basic vectors containing adipolin promoter region (−66/−1 or −111/−1) or empty pGL3 vector in the presence of pRL-SV40. Promoter activity was assessed by luciferase reporter assay. Results are normalized relative to the values of empty pShuttle vectors (MOCK). N = 6 in each group. **C**, Luciferase assay for determination of adipolin promoter activity in 3T3-L1 adipocytes. 3T3-L1 adipocytes were transfected with pGL3-basic vectors containing adipolin promoter (−66/−1 or −111/−1) or empty pGL3 vector in the presence of pRL-SV40. N = 6 in each group.

To examine whether KLF15 or KLF9 modulates promoter activity of adipolin, HEK293 cells were transfected with expression vectors containing FLAG-tagged KLF15 or FLAG-tagged KLF9 cDNA, or mock-transfected along with luciferase reporter vectors containing mouse adipolin promoter. KLF15 or KLF9 protein was detected in the cell lysate of the cells expressing KLF15 or KLF9 by Western blot analysis (Figure 3A and B). Expression of KLF15 significantly increased the luciferase activity at the region between −111 to −1 of adipolin promoter compared with mock transfection (Figure 3A). In contrast, the promoter activity of adipolin (−66/−1) in response to KLF15 was diminished compared with that of the region of −111/−1. On the other hand, expression of KLF9 did not affect the luciferase activities of the promoter regions of −66/−1 and −111/−1 (Figure 3B).

To determine which promoter region of adipolin is activated in adipocytes, 3T3-L1 adipocytes were transfected with luciferase reporter plasmids containing the adipolin promoter (−66/−1 and −111/−1). Enhanced luciferase activity of adipolin promoter (−111/−1) was observed in 3T3-L1 adipocytes (Figure 3C). Little or no activities of adipolin promoter (−66/−1) were observed in adipocytes. These data indicate that the region between −111 and −66 is required for the basal activation of adipolin gene promoter in adipocytes and the promoter activation in response to KLF15. Analysis of sequences of this region revealed GC-rich elements, which are reportedly recognized by KLFs.

### Inhibition of JNK activity blocks TNFα-mediated inhibition of KLF15 and adipolin

**Figure 4:**
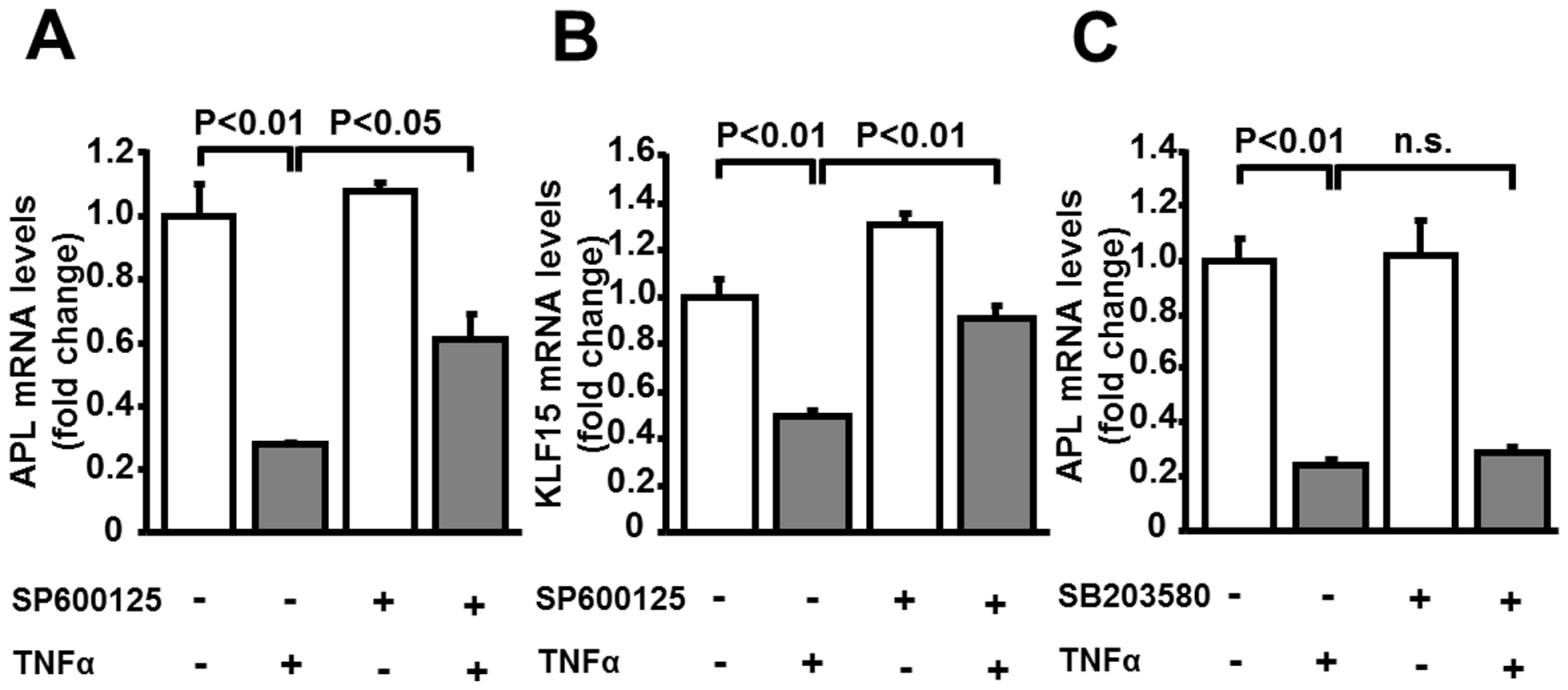
Inhibition of JNK restores TNFα-mediated reduction of adipolin expression in 3T3-L1 adipocytes. The mRNA levels of adipolin (APL) and KLF15 were measured by quantitative RT-PCR method. **A and B**, Effect of JNK inhibitor on mRNA levels of APL (A) and KLF15 (B) in adipocytes. 3T3-L1 adipocytes were cultured in the presence or absence of JNK inhibitor, SP600125 (10 µM) for 1 h followed by treatment with TNFα (10 ng/ml) or vehicle for 24 h. N = 3 in each group. **C**, Effect of p38 MAPK inhibitor on APL mRNA levels in adipocytes. 3T3-L1 adipocytes were treated with or without p38 MAPK inhibitor, SB203580 (10 µM) for 1 h followed by stimulation with TNFα (10 ng/ml) or vehicle for 24 h. N = 3 in each group.

To investigate the mechanism of adipolin suppression by TNFα, 3T3-L1 adipocytes were treated with pharmacological inhibitors of JNK and p38 MAPK which function downstream of TNFα. The JNK inhibitor, SP600125, cancelled the suppressive effects of TNFα on adipolin expression in adipocytes (Figure 4A). SP600125 also restored the reduction of KLF15 expression by TNFα (Figure 4B). SP600125 did not affect the basal expression levels of adipolin and KLF15. In contrast, p38 MAPK inhibitor, SB203580, had no effects on adipolin expression in the presence or absence of TNFα (Figure 4C).

### Ablation of KLF15 reduces adipolin expression in 3T3-L1 adipocytes

**Figure 5:**
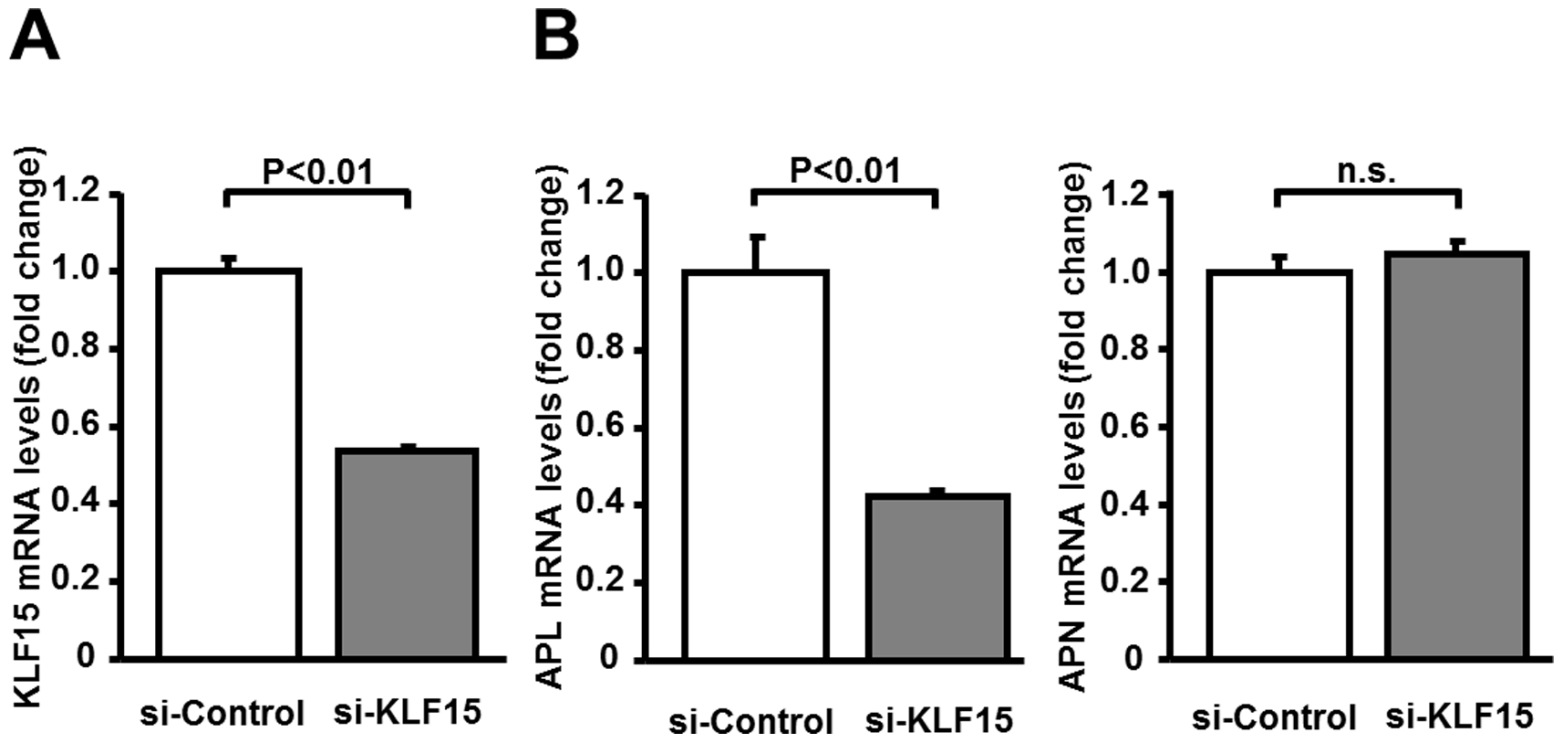
Ablation of KLF15 by siRNA reduces adipolin expression in adipocytes. KLF15, adipolin (APL) and adiponectin (APN) mRNA levels were determined by quantitative RT-PCR method. **A**, KLF15 mRNA levels in 3T3-L1 adipocytes at 48 h after transfection with siRNA targeting KLF15 (si-KLF15) (20 nM) or non-targeting control siRNA (si-Control) (20 nM). N = 3 in each group. **B**, mRNA levels of APL and APN in 3T3-L1 adipocytes transfected with si-KLF15 (20 nM) or si-Control (20 nM). N = 3 in each group.

To examine the role of endogenous KLF15 in control of adipolin expression, 3T3-L1 adipocytes were transfected with siRNA targeting KLF15 (siKLF15) or control siRNA (siControl). Treatment of adipocytes with siKLF15 reduced KLF15 expression by 54% compared to siControl (Figure 5A). Reduction of KLF15 by siKLF15 led to suppression of adipolin expression by 42% compared to siControl (Figure 5B). In contrast, treatment with siKLF15 didn't change the expression levels of adiponectin in adipocytes.

## Discussion

The present study demonstrated that obese states reduce adipolin expression, at least in part, through suppression of KLF15 expression in adipose tissue. Reduced expression of adipolin and KLF15 was observed in obese adipose tissue in vivo and in TNFα-treated cultured adipocytes in vitro. Adenovirus-mediated overexpression of KLF15 restored the suppressive effects of TNFα on adipolin expression in adipocytes. KLF15 stimulated the promoter activity of adipolin in 293 cells. TNF-α-mediated reduction of adipolin and KLF15 expression in adipocytes was reversed by pretreatment with JNK inhibitor. Adipolin acts as an adipokine that exerts beneficial actions on glucose metabolism [17], [18]. These data suggest that the pro-inflammatory responses to obesity can induce JNK-dependent suppression of KLF15-adipolin regulatory axis in adipose tissue, thereby leading to exacerbation of metabolic dysfunction.

Adipolin is present in blood stream as full and proteolytically cleaved isoforms [27], [28]. Obese mice show an increase in cleaved/full adipolin protein ratio in plasma despite reduced plasma levels of full and total (full and cleaved) adipolin compared to lean mice [27]. Furin, which belongs to the member of proprotein convertase family, is involved in proteolytic cleavage of adipolin [27], [28]. Furin expression is up-regulated in adipose tissue of obese mice [27]. Thus, it is conceivable that furin induction under conditions of obesity facilitates the cleavage of adipolin in fat tissue, thereby leading to reduced levels of full forms of circulating adipolin. It has been shown that full, but not cleaved isoform of adipolin enhances insulin-stimulated glucose uptake in cultured adipocytes [28]. Taken together with our present findings, these results suggest that obese states repress the transcript levels of adipolin through reduction of KLF15 expression and concomitantly suppress the production of insulin-sensitizing full form of adipolin protein through induction of furin, therefore contributing to the progression of insulin resistance.

Several KLFs including KLF3, KLF9 and KLF15 are implicated in adipogenesis and metabolic regulation [21], [22], [29]. A recent report showed that KLF3 binds to adipolin promoter, thereby leading to suppression of promoter activity [30]. In addition, adipolin expression are elevated in white adipose tissue and plasma in KLF3 knockout mice [30]. These findings indicate that KLF3 can act as a repressor of adipolin transcription in adipocytes. Our data showed that KLF15 enhances the promoter activity of adipolin gene. Therefore, it is possible that KLF15 shares its target gene adipolin with KLF3, and that KLF15 and KLF3 act as a competitive factor that positively and negatively regulates adipolin transcription. Our observations demonstrate that adipose KLF15 but not KLF3 was down-regulated by obesity or the inflammatory stimulus, suggesting that KLF3 has a minor role in regulation of adipolin expression in fat tissue under conditions of obesity. However, we cannot exclude the possibility that obesity-induced perturbations in the balance between KLF15 and KLF3 expression may affect adipolin expression.

It has been shown that KLF15 increases the expression of insulin-sensitive glucose transporter GLUT4 in adipose and muscle cells [31]. It has also been reported that KLF15 can promote adipogenesis though induction of PPAR-γ [21]. Acetyl-CoA synthetase 2 is shown to be a target of KLF15 in muscle [32]. KLF15 knockout mice display severe hypoglycemia after fasting due to defective amino acid catabolism [33]. Furthermore, adipose specific KLF15 transgenic mice show improved glucose tolerance due to enhanced insulin secretion [34]. Here we showed that KLF15 serves as an activator of adipolin expression in adipocytes. Thus, KLF15 may function as a crucial regulator of glucose homeostasis through modulation of various metabolic-related genes in its target cells.

Inflammatory responses in obese adipose tissue participate in systemic metabolic dysfunction including insulin resistance [4], [35], [36]. TNFα is a major pro-inflammatory adipokine that is upregulated in fat tissue of obese animals and humans, and causes adipose tissue inflammation and insulin resistance in obesity [8]. Indeed, inhibition or deletion of TNFα signaling dramatically improves insulin resistance in rodent models of obesity [8], [37]. Activation of JNK, which is induced by various inflammatory stimuli including TNFα, is involved in the development of obesity-related insulin resistance [38], [39]. In 3T3-L1 adipocytes, TNFα stimulates pro-inflammatory cytokine expression through JNK activation [40]. Furthermore, ablation of JNK in adipose tissue leads to improvement of high fat diet-induced insulin resistance in a mouse model [41]. The present study indicates that JNK activation caused by TNFα attenuates adipolin expression in adipocytes via suppression of KLF15. Thus, inhibition of JNK-dependent inflammatory cascade in obese states may contribute to improved insulin sensitivity partly through enhanced production of adipose adipolin.

Adipolin belongs to the family of CTRPs, which are conserved adiponectin paralogs [17], [18]. Both adiponectin and adipolin are down-regulated in fat depot and blood stream by obesity [17], [18], [23]. Likewise, treatment of adipocytes with TNFα leads to reduction of adiponectin and adipolin expression. Previous reports showed that PPAR-γ agonist enhances adiponectin gene expression in cultured adipocytes and adipose tissue, resulting in increased levels of circulating adiponectin [23], [42], [43]. Moreover, PPAR-γ agonist reverses TNFα-induced suppression of adiponectin expression in adipocytes. Although KLF15 induces PPAR-γ expression during adipocyte differentiation [21], our data showed that knockdown of KLF15 has no effects on adiponectin expression in adipocytes. In addition, KLF15 marginally increased adiponectin expression in adipocytes but had little effects on TNFα-induced down-regulation of adiponectin. Moreover, adipose specific KLF15 transgenic mice have been reported to exhibit rather lower serum levels of adiponectin than control littermates (35). Thus, it is unlikely that KLF15 acts as a major determinant of adiponectin expression.

### Conclusion

We demonstrated for the first time that KLF15 acts as a novel regulator of adipolin expression in adipocytes and that the pro-inflammatory states caused by obesity lead to reduction of adipose adipolin expression via suppression of KLF15 activation. Strategies to normalize or enhance adipolin production by targeting KLF15 can be valuable for the treatment or prevention of metabolic dysfunction in obesity.

## References

[1] Friedman JM (2003) A war on obesity, not the obese. Science 299: 856–858. 12574619 10.1126/science.1079856

[2] Matsuzawa Y (2006) Therapy Insight: adipocytokines in metabolic syndrome and related cardiovascular disease. Nat Clin Pract Cardiovasc Med 3: 35–42. 16391616 10.1038/ncpcardio0380

[3] Despres JP, Lemieux I (2006) Abdominal obesity and metabolic syndrome. Nature 444: 881–887. 17167477 10.1038/nature05488

[4] Neels JG, Olefsky JM (2006) Inflamed fat: what starts the fire? J Clin Invest 116: 33–35. 16395402 10.1172/JCI27280 PMC1323268

[5] Ouchi N, Parker JL, Lugus JJ, Walsh K (2011) Adipokines in inflammation and metabolic disease. Nat Rev Immunol 11: 85–97. 21252989 10.1038/nri2921 PMC3518031

[6] Donath MY, Shoelson SE (2011) Type 2 diabetes as an inflammatory disease. Nat Rev Immunol 11: 98–107. 21233852 10.1038/nri2925

[7] Moore KJ, Tabas I (2011) Macrophages in the pathogenesis of atherosclerosis. Cell 145: 341–355. 21529710 10.1016/j.cell.2011.04.005 PMC3111065

[8] Hotamisligil GS, Shargill NS, Spiegelman BM (1993) Adipose expression of tumor necrosis factor-alpha: direct role in obesity-linked insulin resistance. Science 259: 87–91. 7678183 10.1126/science.7678183

[9] Maeda K, Okubo K, Shimomura I, Funahashi T, Matsuzawa Y, et al (1996) cDNA cloning and expression of a novel adipose specific collagen-like factor, apM1 (AdiPose Most abundant Gene transcript 1). Biochem Biophys Res Commun 221: 286–289. 8619847 10.1006/bbrc.1996.0587

[10] Maeda N, Shimomura I, Kishida K, Nishizawa H, Matsuda M, et al (2002) Diet-induced insulin resistance in mice lacking adiponectin/ACRP30. Nat Med 8: 731–737. 12068289 10.1038/nm724

[11] Ouchi N, Kihara S, Funahashi T, Matsuzawa Y, Walsh K (2003) Obesity, adiponectin and vascular inflammatory disease. Curr Opin Lipidol 14: 561–566. 14624132 10.1097/00041433-200312000-00003

[12] Takemura Y, Ouchi N, Shibata R, Aprahamian T, Kirber MT, et al (2007) Adiponectin modulates inflammatory reactions via calreticulin receptor-dependent clearance of early apoptotic bodies. J Clin Invest 117: 375–386. 17256056 10.1172/JCI29709 PMC1770947

[13] Ouchi N, Kihara S, Arita Y, Maeda K, Kuriyama H, et al (1999) Novel modulator for endothelial adhesion molecules: adipocyte-derived plasma protein adiponectin. Circulation 100: 2473–2476. 10604883 10.1161/01.cir.100.25.2473

[14] Ohashi K, Parker JL, Ouchi N, Higuchi A, Vita JA, et al (2010) Adiponectin promotes macrophage polarization toward an anti-inflammatory phenotype. J Biol Chem 285: 6153–6160. 20028977 10.1074/jbc.M109.088708 PMC2825410

[15] Ohashi K, Kihara S, Ouchi N, Kumada M, Fujita K, et al (2006) Adiponectin replenishment ameliorates obesity-related hypertension. Hypertension 47: 1108–1116. 16651465 10.1161/01.HYP.0000222368.43759.a1

[16] Shibata R, Sato K, Pimentel DR, Takemura Y, Kihara S, et al (2005) Adiponectin protects against myocardial ischemia-reperfusion injury through AMPK- and COX-2-dependent mechanisms. Nat Med 11: 1096–1103. 16155579 10.1038/nm1295 PMC2828682

[17] Enomoto T, Ohashi K, Shibata R, Higuchi A, Maruyama S, et al (2011) Adipolin/C1qdc2/CTRP12 protein functions as an adipokine that improves glucose metabolism. J Biol Chem 286: 34552–34558. 21849507 10.1074/jbc.M111.277319 PMC3186379

[18] Wei Z, Peterson JM, Wong GW (2011) Metabolic regulation by C1q/TNF-related protein-13 (CTRP13): activation OF AMP-activated protein kinase and suppression of fatty acid-induced JNK signaling. J Biol Chem 286: 15652–15665. 21378161 10.1074/jbc.M110.201087 PMC3091174

[19] Pearson R, Fleetwood J, Eaton S, Crossley M, Bao S (2008) Kruppel-like transcription factors: a functional family. Int J Biochem Cell Biol 40: 1996–2001. 17904406 10.1016/j.biocel.2007.07.018

[20] McConnell BB, Yang VW (2010) Mammalian Kruppel-like factors in health and diseases. Physiol Rev 90: 1337–1381. 20959618 10.1152/physrev.00058.2009 PMC2975554

[21] Mori T, Sakaue H, Iguchi H, Gomi H, Okada Y, et al (2005) Role of Kruppel-like factor 15 (KLF15) in transcriptional regulation of adipogenesis. J Biol Chem 280: 12867–12875. 15664998 10.1074/jbc.M410515200

[22] Pei H, Yao Y, Yang Y, Liao K, Wu JR (2011) Kruppel-like factor KLF9 regulates PPARgamma transactivation at the middle stage of adipogenesis. Cell Death Differ 18: 315–327. 20725087 10.1038/cdd.2010.100 PMC3131894

[23] Maeda N, Takahashi M, Funahashi T, Kihara S, Nishizawa H, et al (2001) PPARgamma ligands increase expression and plasma concentrations of adiponectin, an adipose-derived protein. Diabetes 50: 2094–2099. 11522676 10.2337/diabetes.50.9.2094

[24] Kilroy G, Burk DH, Floyd ZE (2009) High efficiency lipid-based siRNA transfection of adipocytes in suspension. PLoS One 4: e6940. 19759827 10.1371/journal.pone.0006940 PMC2736387

[25] Ouchi N, Oshima Y, Ohashi K, Higuchi A, Ikegami C, et al (2008) Follistatin-like 1, a secreted muscle protein, promotes endothelial cell function and revascularization in ischemic tissue through a nitric-oxide synthase-dependent mechanism. J Biol Chem 283: 32802–32811. 18718903 10.1074/jbc.M803440200 PMC2583310

[26] Shibata R, Ouchi N, Kihara S, Sato K, Funahashi T, et al (2004) Adiponectin stimulates angiogenesis in response to tissue ischemia through stimulation of amp-activated protein kinase signaling. J Biol Chem 279: 28670–28674. 15123726 10.1074/jbc.M402558200

[27] Enomoto T, Shibata R, Ohashi K, Kambara T, Kataoka Y, et al (2012) Regulation of adipolin/CTRP12 cleavage by obesity. Biochem Biophys Res Commun 428: 155–159. 23068097 10.1016/j.bbrc.2012.10.031

[28] Wei Z, Lei X, Seldin MM, Wong GW (2012) Endopeptidase cleavage generates a functionally distinct isoform of C1q/tumor necrosis factor-related protein-12 (CTRP12) with an altered oligomeric state and signaling specificity. J Biol Chem 287: 35804–35814. 22942287 10.1074/jbc.M112.365965 PMC3476250

[29] Sue N, Jack BH, Eaton SA, Pearson RC, Funnell AP, et al (2008) Targeted disruption of the basic Kruppel-like factor gene (Klf3) reveals a role in adipogenesis. Mol Cell Biol 28: 3967–3978. 18391014 10.1128/MCB.01942-07 PMC2423134

[30] Bell-Anderson KS, Funnell AP, Williams H, Mat Jusoh H, Scully T, et al (2013) Loss of Kruppel-Like Factor 3 (KLF3/BKLF) Leads to Upregulation of the Insulin-Sensitizing Factor Adipolin (FAM132A/CTRP12/C1qdc2). Diabetes 62: 2728–2737. 23633521 10.2337/db12-1745 PMC3717849

[31] Gray S, Feinberg MW, Hull S, Kuo CT, Watanabe M, et al (2002) The Kruppel-like factor KLF15 regulates the insulin-sensitive glucose transporter GLUT4. J Biol Chem 277: 34322–34328. 12097321 10.1074/jbc.M201304200

[32] Yamamoto J, Ikeda Y, Iguchi H, Fujino T, Tanaka T, et al (2004) A Kruppel-like factor KLF15 contributes fasting-induced transcriptional activation of mitochondrial acetyl-CoA synthetase gene AceCS2. J Biol Chem 279: 16954–16962. 14960588 10.1074/jbc.M312079200

[33] Gray S, Wang B, Orihuela Y, Hong EG, Fisch S, et al (2007) Regulation of gluconeogenesis by Kruppel-like factor 15. Cell Metab 5: 305–312. 17403374 10.1016/j.cmet.2007.03.002 PMC1892530

[34] Nagare T, Sakaue H, Matsumoto M, Cao Y, Inagaki K, et al (2011) Overexpression of KLF15 transcription factor in adipocytes of mice results in down-regulation of SCD1 protein expression in adipocytes and consequent enhancement of glucose-induced insulin secretion. J Biol Chem 286: 37458–37469. 21862590 10.1074/jbc.M111.242651 PMC3199492

[35] Xu H, Barnes GT, Yang Q, Tan G, Yang D, et al (2003) Chronic inflammation in fat plays a crucial role in the development of obesity-related insulin resistance. J Clin Invest 112: 1821–1830. 14679177 10.1172/JCI19451 PMC296998

[36] Lumeng CN, Bodzin JL, Saltiel AR (2007) Obesity induces a phenotypic switch in adipose tissue macrophage polarization. J Clin Invest 117: 175–184. 17200717 10.1172/JCI29881 PMC1716210

[37] Uysal KT, Wiesbrock SM, Marino MW, Hotamisligil GS (1997) Protection from obesity-induced insulin resistance in mice lacking TNF-alpha function. Nature 389: 610–614. 9335502 10.1038/39335

[38] Hirosumi J, Tuncman G, Chang L, Gorgun CZ, Uysal KT, et al (2002) A central role for JNK in obesity and insulin resistance. Nature 420: 333–336. 12447443 10.1038/nature01137

[39] Ouchi N, Higuchi A, Ohashi K, Oshima Y, Gokce N, et al (2010) Sfrp5 is an anti-inflammatory adipokine that modulates metabolic dysfunction in obesity. Science 329: 454–457. 20558665 10.1126/science.1188280 PMC3132938

[40] Kawamata Y, Imamura T, Babendure JL, Lu JC, Yoshizaki T, et al (2007) Tumor necrosis factor receptor-1 can function through a G alpha q/11-beta-arrestin-1 signaling complex. J Biol Chem 282: 28549–28556. 17664271 10.1074/jbc.M705869200

[41] Sabio G, Das M, Mora A, Zhang Z, Jun JY, et al (2008) A stress signaling pathway in adipose tissue regulates hepatic insulin resistance. Science 322: 1539–1543. 19056984 10.1126/science.1160794 PMC2643026

[42] Kubota N, Terauchi Y, Kubota T, Kumagai H, Itoh S, et al (2006) Pioglitazone ameliorates insulin resistance and diabetes by both adiponectin-dependent and -independent pathways. J Biol Chem 281: 8748–8755. 16431926 10.1074/jbc.M505649200

[43] Pereira RI, Leitner JW, Erickson C, Draznin B (2008) Pioglitazone acutely stimulates adiponectin secretion from mouse and human adipocytes via activation of the phosphatidylinositol 3'-kinase. Life Sci 83: 638–643. 18824177 10.1016/j.lfs.2008.09.002

